# Supplementary figures and images for: Dissecting the Specificity of Protein-Protein Interaction in Bacterial Two-Component Signaling: Orphans and Crosstalks
Source: PLoS One. 2011 May 9;6(5):e19729. doi: 10.1371/journal.pone.0019729 (PMC3090404; doi:10.1371/journal.pone.0019729)

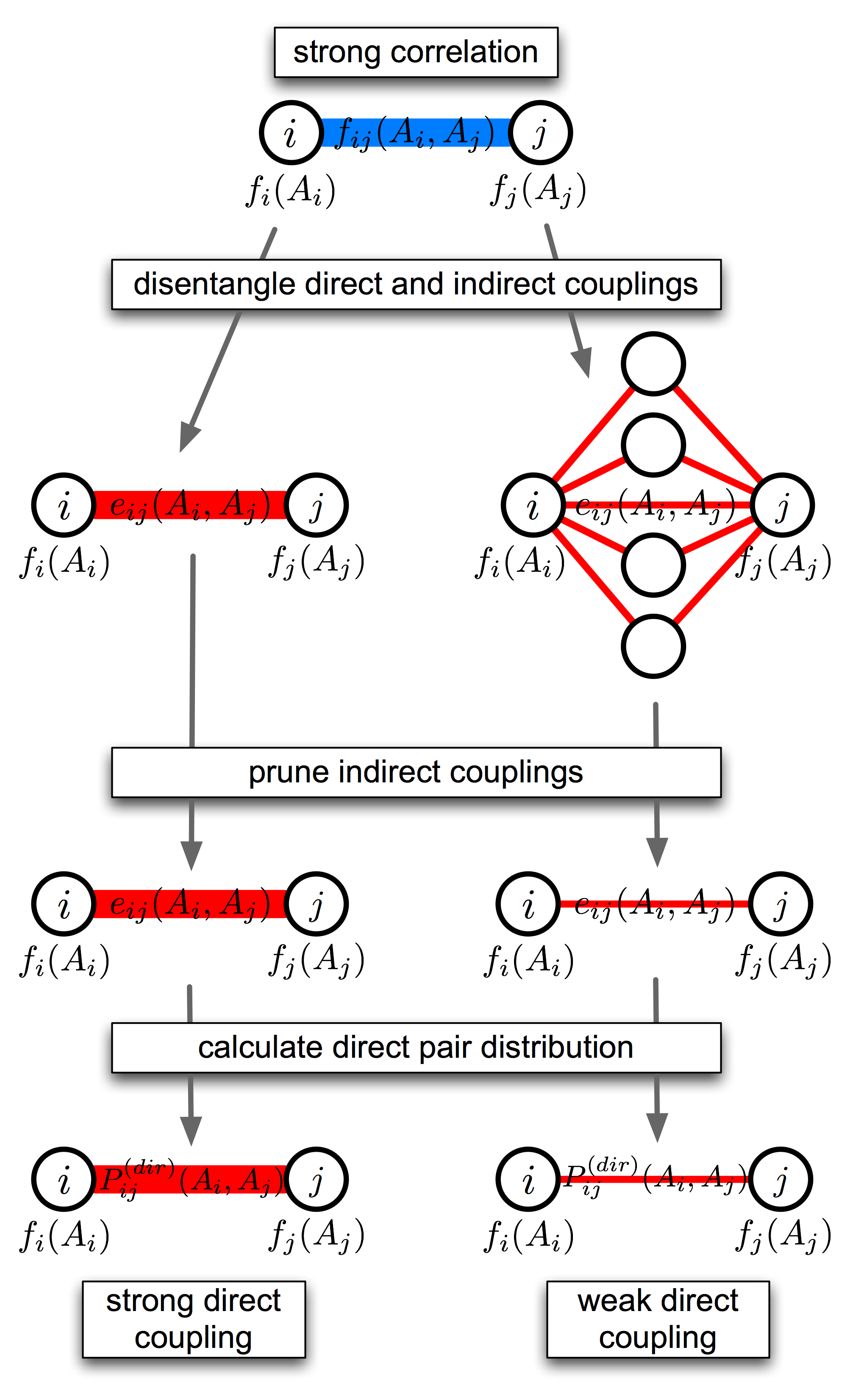

Supplement: Figure S1 — From pair frequency counts to direct pair distributions. The figure shows a schematic description of the Direct Coupling Analysis (DCA). Strong correlation of the amino-acid occupation of two MSA columns i and j (detected by mutual information) may result from two different scenarios (and any mixture of the two): The two positions have a strong direct statistical coupling, or their correlation results from indirect couplings via intermediate positions. DCA disentangles direct and indirect couplings. Pruning all indirect couplings, one can determine the coupling matrices eij(A,B) and the direct pair distributions , which are used in the scoring function. Technical details are explained in Weigt, M, et al. (2009) Proc Natl Acad Sci USA 106: 67–72. (TIFF) [file pone.0019729.s001.tif]

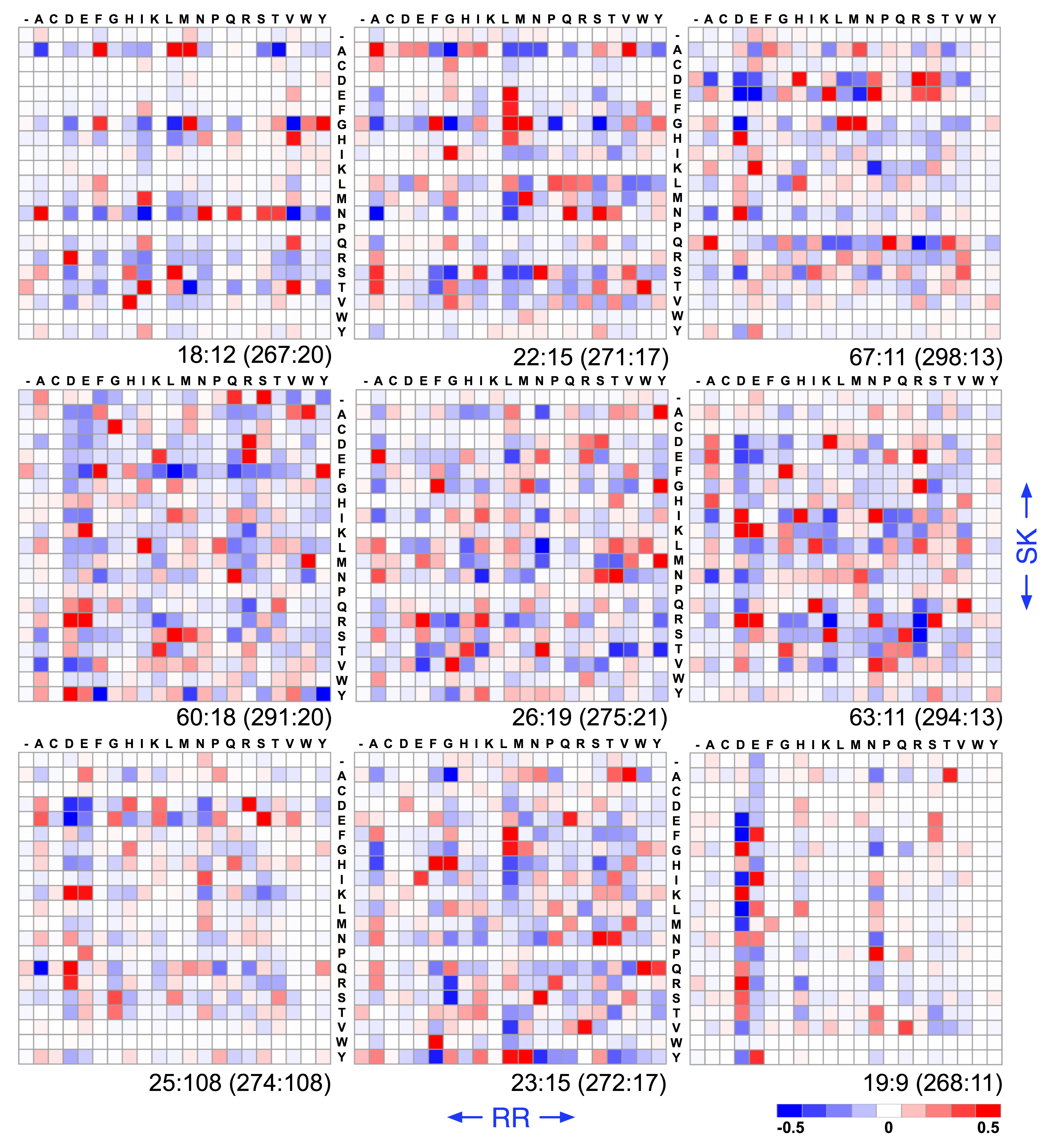

Supplement: Figure S2 — Individual coupling matrices for the 9 strongest direct couplings between SK and RR positions. The figures number all positions according to the Pfam HMMs and, in parenthesis, to the structural template HK853/RR468 (PDB ID: 3dge, Casino, P. et al. (2009) Cell 139: 325–336). Note that 25:108 was not contained in the original DCA paper (Weigt, M, et al. (2009) Proc Natl Acad Sci 106: 67–72) but showed up only in the larger cognate MSA used here. It includes the RR position 108, which, along the sequence, is far from the other identified RR interface positions. The contact 25:108 is made (3.0 Å minimal atom distance) in HK853/RR468. (TIFF) [file pone.0019729.s002.tif]

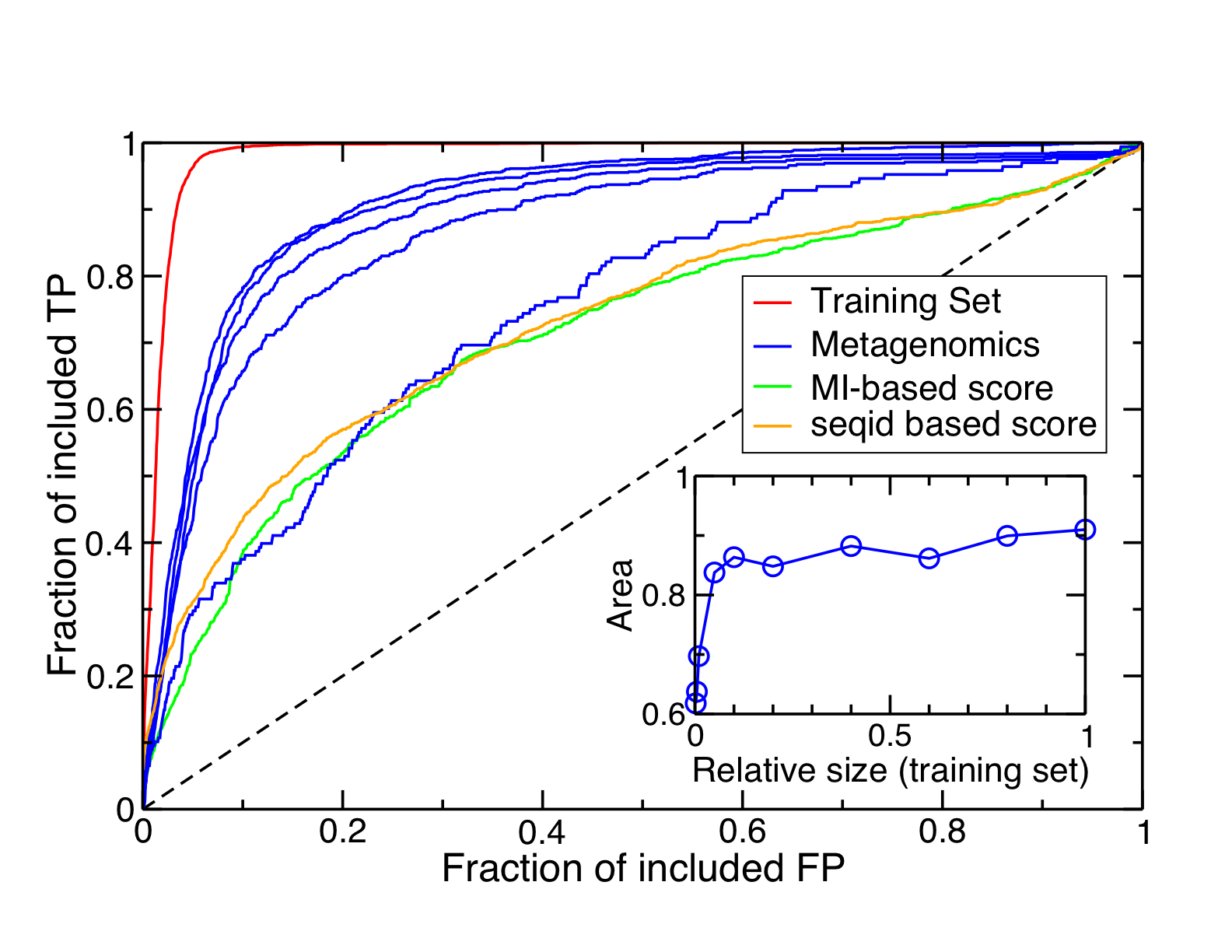

Supplement: Figure S3 — Prediction accuracy of the scoring function for metagenomic two-component system pairings. ROC curves for the genomic training set (red) and the metagenomic test set (blue), as ordered by the interaction score. The different blue curves correspond to different subsets of the metagenomic data, which are filtered according to their dissimilarity from all genomic TCS (from top to bottom: all metagenomic TCS, metagenomic TCS featuring less than 80%, 70%, 60% resp. 50% sequence identity with all genomic TCS). The green curve is the ROC curve for the full metagenomic test set using the purely MI-based score suggested in (White, R. A. et al. (2007) Methods Enzymol 422, 75–101), the orange curve for a kNN-type scoring scheme based purely on % sequence identities (k = 2). The inset shows the size of the area under the metagenomic ROC curve as a function of the size of the training set (relative number of included species). (TIFF) [file pone.0019729.s003.tif]

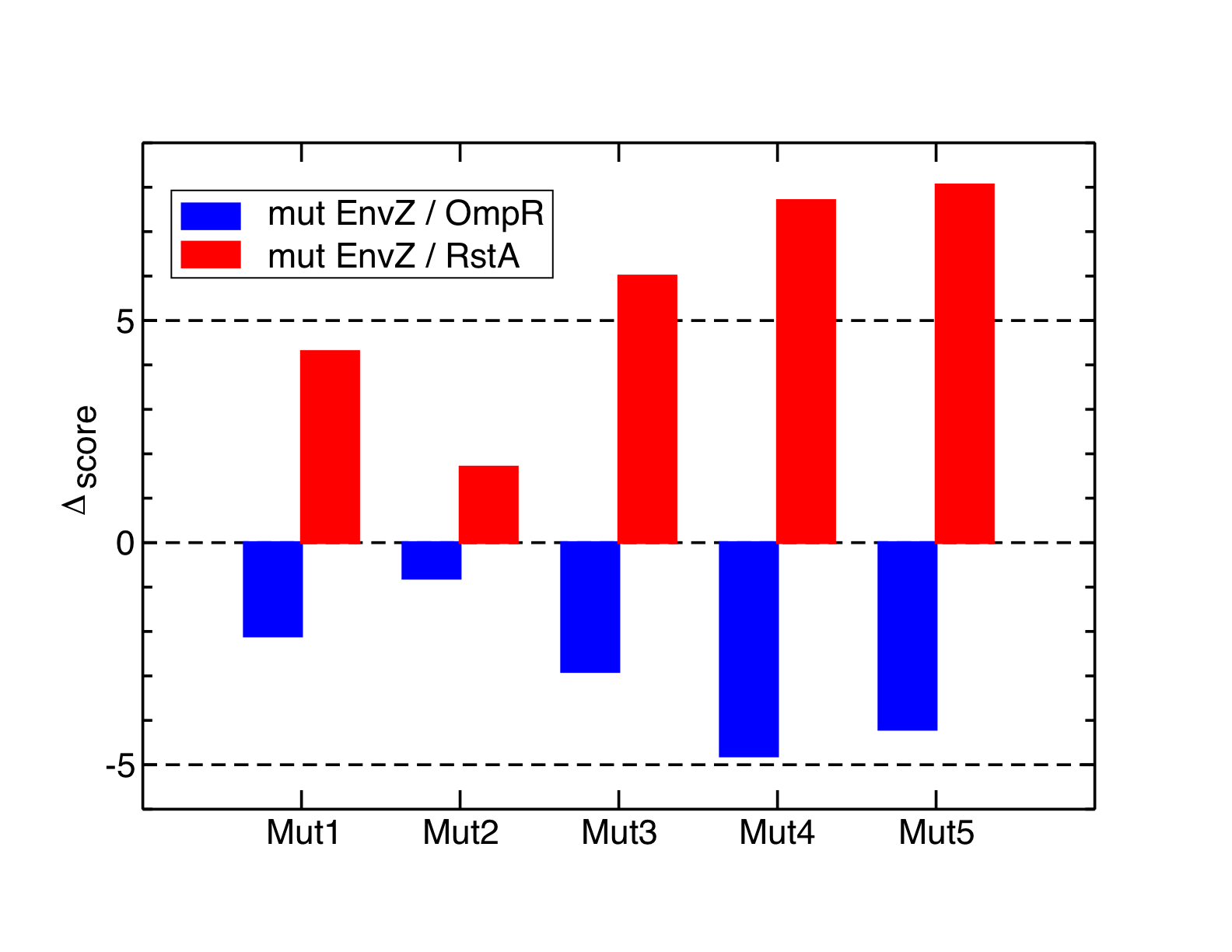

Supplement: Figure S4 — Effect of point mutations on sensor kinase/response regulator scores. Score differences between the SK EnvZ mutants considered by (Skerker et al. (2008) Cell 133, 1043–1054) and WT EnvZ, against its native interaction partner RR OmpR and another RR RstA. The individual mutations were Mut1: L254Y; Mut2: A255R; Mut3: L254Y/A255R; Mut4: T250V/L254Y/A255R, Mut5: T250V/L254Y/A255R/S269A. Experiments by Skerker et al. demonstrated that Mut1–3 were promiscuous, phosphorylating OmpR and RstA with similar kinetics, whereas Mut4–5 preferred RR RstA over the native partner OmpR. The figure displays the score differences ( = mutant score – WT score) for these substitutions. Scores of EnvZ with OmpR are found to be generally decreasing with the number of mutations (blue bars), whereas scores with RstA generally increase. This result illustrates that the scoring function is able to qualitatively capture the influence of single- and multiple- site substitutions. It is however not yet able to predict the point where specificity switches from the original cognate RR toward the new partner. (TIFF) [file pone.0019729.s004.tif]

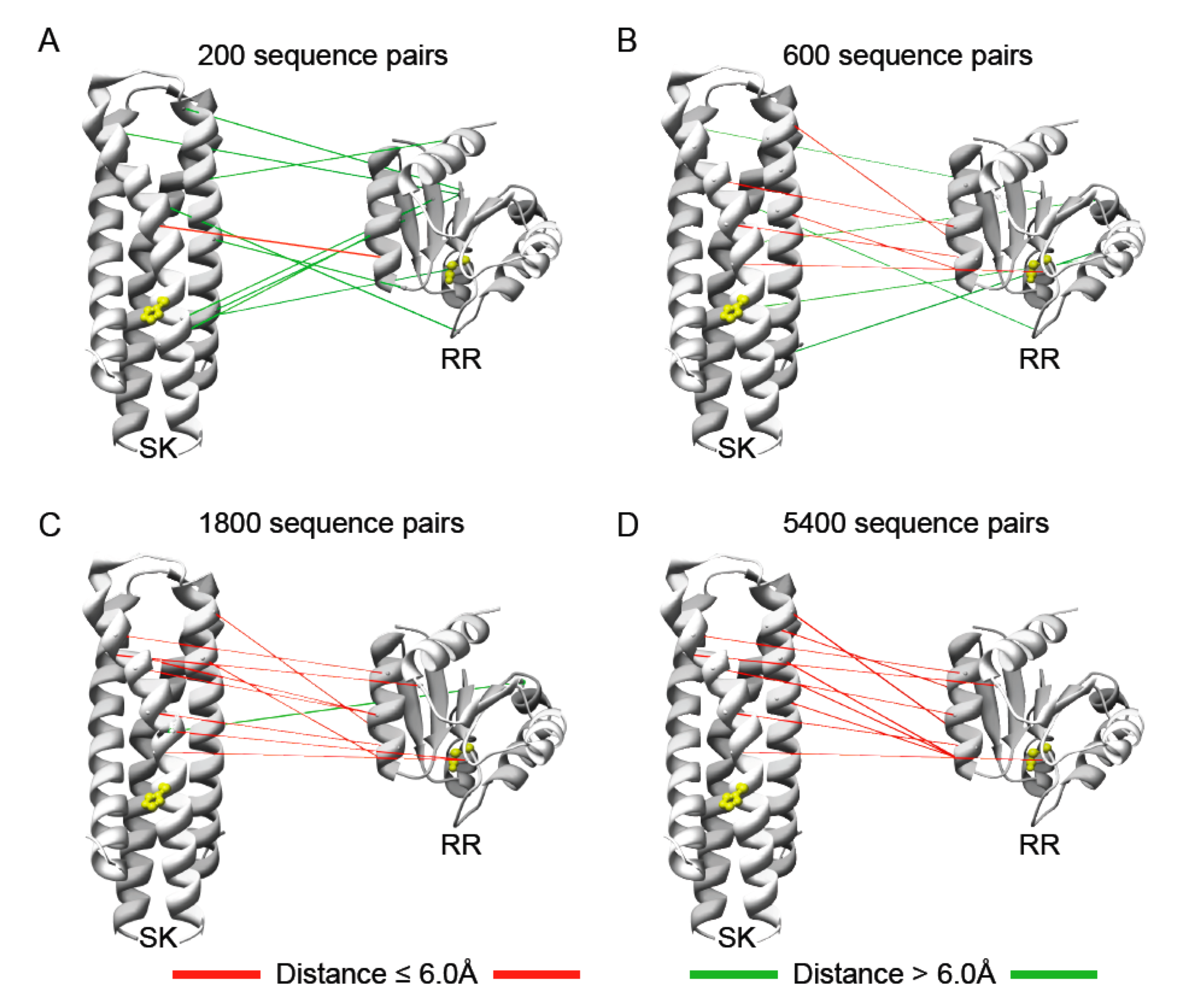

Supplement: Figure S5 — Inference of directly coupled residue pairs from reduced data sets. Shown are the 10 strongest directly coupled residue pairs between SK and RR, for 200 (panel A), 600 (panel B), 1800 (panel C) resp. 5400 (panel D) randomly selected cognate SK/RR sequences. Residue pairs in contact are shown in red, distant pairs in green. The figure illustrates the degradation of the signal in case of insufficient sequence statistics. (TIFF) [file pone.0019729.s005.tif]
