# Supplementary material for: Dissecting the Specificity of Protein-Protein Interaction in Bacterial Two-Component Signaling: Orphans and Crosstalks
Source: PLoS One. 2011 May 9;6(5):e19729. doi: 10.1371/journal.pone.0019729 (PMC3090404; doi:10.1371/journal.pone.0019729)
Supplement: Text S1 — Testing the score on metagenomic data. The predictive performance of the scoring function is tested using cognate TCS extracted from metagenomic databases. (DOC) [file pone.0019729.s008.doc]

**Supplemental Information:**

**Dissecting the Specificity of Protein-Protein Interaction in Bacterial Two-Component Signaling: Orphans and Crosstalks**

Andrea Procaccini, Bryan Lunt, Hendrik Szurmant, Terence Hwa, Martin Weigt

**Testing the score on metagenomic data**

To test the scoring function on TCS which were not used for deriving the statistical model Eq. (5), we considered metagenomic data to increase the number of available interacting protein pairs beyond the ones provided by genomic data sets. Currently, the length of sequenced DNA pieces (contigs) is very restricted in metagenomics, typical lengths are of 1-2 kbp per contig. As a consequence, the current availability of entire operons is very restricted, as compared to the total number of proteins found in metagenomics. We have scanned two major metagenomic databases (DOE [http://img.jgi.doe.gov](http://img.jgi.doe.gov/), CAMERA [http://camera.calit2.net](http://camera.calit2.net/)) and extracted 1446 cognate SK/RR pairs, which are used as test sets for the scoring function derived from genomic data. 1,243 (920, 589, 168) of these sequences have less than 80% (70%, 60%, 50%) sequence identity to all 8,998 genomic cognate TCS.

The scoring function Eq. (9) (inferred using only genomic data) was used to test each of the 1446x1446 possible SK/RR pairings, and these pairings were ordered according to the obtained score. In order to estimate true-positive (TP) and false-positive (FP) rates, all 1446 SK/RR pairs on the same contig are counted as positives (P), and all pairs on different contigs as negatives (N). Fig. S3 shows the ROC curve (receiver operating characteristic curve, upper blue curve), i.e. the ratio TP/P (the fraction of all P which are included as TP) as a function of the ratio FP/N (the fraction of all N which are included as FP) when more and more SK/RR pairs are included going down their score-ordered list. The area under this curve is 91%; a random prediction would give 50%, a perfect prediction 100%. These numbers illustrate the predictive power of our scoring system. The result is rather robust with respect to changes in the size of the MSA used for constructing the score: We have randomly selected a fraction of the available genomes, assembled all cognate SK/RR belonging to these genomes, recalculated the scoring function based on the restricted MSA, and reanalyzed the metagenomic data. The inset of Fig. S3 reports the area under the ROC curve as a function of the fraction of considered genomes. Already ca. 10% of all 769 genomes lead to an area of more than 85% under the ROC curve.

Some of the meta-genomic TCS are actually contained in or very similar to the genomic cognate TCS. To obtain a more strict evaluation of our scoring function, we have restricted the metagenomic sequences to those, which are “sufficiently far” from all 8998 genomic cognate TCS used for constructing the score. We found that 1243, 920, 589 resp. 168 out of the 1446 metagenomic TCS do not possess any genomic partner TCS at more than 80%, 70%, 60% resp. 50% sequence identity, serving therefore as more and more severe testing sets for our scoring system. We find the area under the ROC curve to be relatively stable, i.e. in this order 90%, 89%, 87% resp. 75%, cf. Fig. S3 (blue curves, going from upper to lower curves). Only the very last data set of the 168 metagenomic TCS, which feature less then 50% sequence identity to all genomic TCS, shows a substantial loss of predictive power. We conclude that the genomic cognate TCS do not yet completely sample the space of all theoretically possible TCS, and less-sampled regions are not as well-described by the score as well-sampled regions.

This raises the immediate question, if our scoring system is actually providing information going beyond sequence similarity. To check this, we have tested a kNN type scoring system: For each (intra- and inter-contig) pairing of a metagenomic SK with a metagenomic RR, we have determined the $k,k=1,2,...,20,$ genomic TCS of highest sequence identity, and used the average of these $k$ identity values as a score. The best resulting ROC curve ($k=2$, orange in Fig. S3) has an area of only 73%, illustrating the substantial gain obtained by applying the covariance-based score. Fig. S3 shows also the importance of disentangling direct and indirect correlations using DCA: The green ROC curve results from the purely mutual-information (MI) based score proposed previously in (White, R. A., Szurmant, H., Hoch, J. A. & Hwa, T. (2007) Features of protein-protein interactions in two-component signaling deduced from genomic libraries. *Methods Enzymol* **422,** 75-101); here the area under this curve is 71% and thus comparable to the similarity based score.

All SK/RR pairs on different contigs were considered as negatives, i.e. non-interacting, even if inter-contig interactions are likely to exist (for instance for orthologous signaling systems found on different contigs). It is thus to be expected that the scoring function performs even better than reflected by the above values. The conjecture that the scoring system actually identifies rare but existing cross-contig SK/RR interactions, will be tested in detail in the next section in the context of crosstalk between pairs of cognate SK/RR systems.
